# Prediction of postoperative health-related quality of life among patients with metastatic spinal cord compression secondary to lung cancer

**DOI:** 10.3389/fendo.2023.1206840

**Published:** 2023-09-01

**Authors:** Yufang Fu, Weiqing Shi, Jing Zhao, Xuyong Cao, Yuncen Cao, Mingxing Lei, Xiuyun Su, Qiu Cui, Yaosheng Liu

**Affiliations:** ^1^ Department of Oncology, The Fifth Medical Center of Chinese PLA General Hospital, Beijing, China; ^2^ Department of Operation Room, The Fifth Medical Center of Chinese PLA General Hospital, Beijing, China; ^3^ Chinese PLA Medical School, Beijing, China; ^4^ Senior Department of Orthopedics, The Fourth Medical Center of Chinese PLA General Hospital, Beijing, China; ^5^ Department of Orthopedic Surgery, Hainan Hospital of Chinese PLA General Hospital, Sanya, China; ^6^ National Clinical Research Center for Orthopedics, Sports Medicine and Rehabilitation, Chinese PLA General Hospital, Beijing, China; ^7^ Intelligent Medical Innovation Institute, Southern University of Science and Technology Hospital, Shenzhen, China; ^8^ Department of Orthopedic Surgery, The Fifth Medical Center of Chinese PLA General Hospital, Beijing, China

**Keywords:** lung cancer, metastatic spinal cord compression, health-related quality of life, risk stratification, prediction model

## Abstract

**Background:**

Health-related quality of life (HRQoL) is a critical aspect of overall well-being for patients with lung cancer, particularly those with metastatic spinal cord compression (MSCC). However, there is currently a lack of universal evaluation of HRQoL in this specific patient population. The aim of this study was to develop a nomogram that can accurately predict HRQoL outcomes in patients with lung cancer-related MSCC.

**Methods:**

A total of 119 patients diagnosed with MSCC secondary to lung cancer were prospectively collected for analysis in the study. The least absolute shrinkage and selection operator (LASSO) regression analysis, along with 10-fold cross-validation, was employed to select the most significant variables for inclusion in the nomogram. Discriminative and calibration abilities were assessed using the concordance index (C-index), discrimination slope, calibration plots, and goodness-of-fit tests. Net reclassification index (NRI) and integrated discrimination improvement (IDI) analyses were conducted to compare the nomogram’s performance with and without the consideration of comorbidities.

**Results:**

Four variables were selected to construct the final nomogram, including the Eastern Cooperative Oncology Group (ECOG) score, targeted therapy, anxiety scale, and number of comorbidities. The C-index was 0.87, with a discrimination slope of 0.47, indicating a favorable discriminative ability. Calibration plots and goodness-of-fit tests revealed a high level of consistency between the predicted and observed probabilities of poor HRQoL. The NRI (0.404, 95% CI: 0.074–0.734, p = 0.016) and the IDI (0.035, 95% CI: 0.004–0.066, p = 0.027) confirmed the superior performance of the nomogram with the consideration of comorbidities.

**Conclusions:**

This study develops a prediction nomogram that can assist clinicians in evaluating postoperative HRQoL in patients with lung cancer-related MSCC. This nomogram provides a valuable tool for risk stratification and personalized treatment planning in this specific patient population.

## Introduction

Lung cancer poses a substantial burden on society, ranking second in terms of incidence and first in terms of mortality among all cancer types globally (1). In 2020 alone, there were approximately 2.2 million new cases of lung cancer, accounting for roughly 11.4% of all new cancer cases (1). Tragically, lung cancer remains the leading cause of cancer-related deaths, with an estimated 1.8 million deaths reported in 2020 according to Global Cancer Statistics (1). These figures highlight the alarming reality that over 80% of individuals diagnosed with lung cancer face a grim prognosis, leading to premature death. Moreover, the incidence of lung cancer is steadily increasing worldwide, particularly in developing countries (2), and is projected to double in 2040 (1).

For patients with metastatic spinal cord disease secondary to lung cancer, their condition represents an advanced stage of the disease (3). Those patients often experience severe back pain, neurological complications, and disability (4), all of which significantly impact their health-related quality of life (HRQoL). Furthermore, the therapeutic interventions employed to combat metastatic lung cancer can further impair HRQoL due to associated side effects (5–7), compounded by mental health distress (8). The ongoing COVID-19 pandemic has unleashed unprecedented challenges on society, affecting all aspects of life. Lockdown measures and self-isolation have resulted in income loss and unemployment among cancer patients and their families, leading to emotional issues, financial strain, and further deterioration of HRQoL (9). Consequently, assessing HRQoL in lung cancer patients has never been more critical, particularly amidst the COVID-19 pandemic. Developing a prediction model capable of forecasting and stratifying patients’ HRQoL would greatly assist healthcare professionals in tailoring therapeutic strategies and delivering individualized care. It would also empower patients with crucial HRQoL information, facilitating shared decision-making.

However, to date, postoperative HRQoL outcome remains relatively understudied (10), and no prediction model exists particularly for assessing the HRQoL of lung cancer patients with metastatic spinal disease. Nomograms have emerged as a widely utilized tool for predicting clinical outcomes in cancer patients (11–13). Providing an integrated approach, nomograms are instrumental in personalized medicine and have the potential to become routine tools in clinical practice (14, 15). Therefore, the primary objective of this study was to establish and validate a nomogram specifically designed to predict HRQoL in lung cancer patients, particularly those with metastatic spinal cord compression (MSCC).

## Methods

### Patients

Between April 2019 and November 2022, 119 patients with MSCC from lung cancer were prospectively collected and enrolled at our hospital. Patients’ demographics, received treatments, tumor status, comorbidities, mental health status, and HRQoL were collected and analyzed in the study. The inclusive criteria were carefully established to ensure the selection of suitable participants for this study. First, patients were required to have a confirmed diagnosis of lung cancer through the use of tissue biopsy. Additionally, the criteria demanded that patients exhibit MSCC, which could be verified through either tissue biopsy or various radiographic imaging techniques, such as radionuclide bone scan, magnetic resonance imaging, or computed tomography. Patients experienced corresponding symptoms, such as back pain or neurological disorders, resulting from the metastatic spinal disease. Conversely, certain exclusion criteria were implemented to maintain the integrity and reliability of the study. First, patients who were 20 years of age or younger were excluded to maintain a focus on the adult population affected by this condition. Furthermore, individuals who expressed a lack of willingness to participate were ineligible to ensure a committed and engaged study group. Patients with psychiatric disorders that hindered their ability to cooperate with healthcare professionals were also excluded, as their level of collaboration is crucial for accurate data collection. Similarly, individuals who displayed unconsciousness or profound impairment of self-identity, spatial awareness, temporal perception, and expression of well-being were deemed unfit for the study. Additionally, patients with a predicted life expectancy of less than 3 months were excluded, as their ability to contribute meaningful data over the study period would be limited. Finally, patients who were lost to follow-up were not considered, as their absence from the study would result in incomplete data. The patient’s flowchart is provided in **Supplementary Figure 1**. The study protocol was approved by the Ethics Committee of our hospital and informed written consent was obtained from all patients. The data were all anonymous, and the study complied with the Declaration of Helsinki.

### Surgery

**Figure 1:**
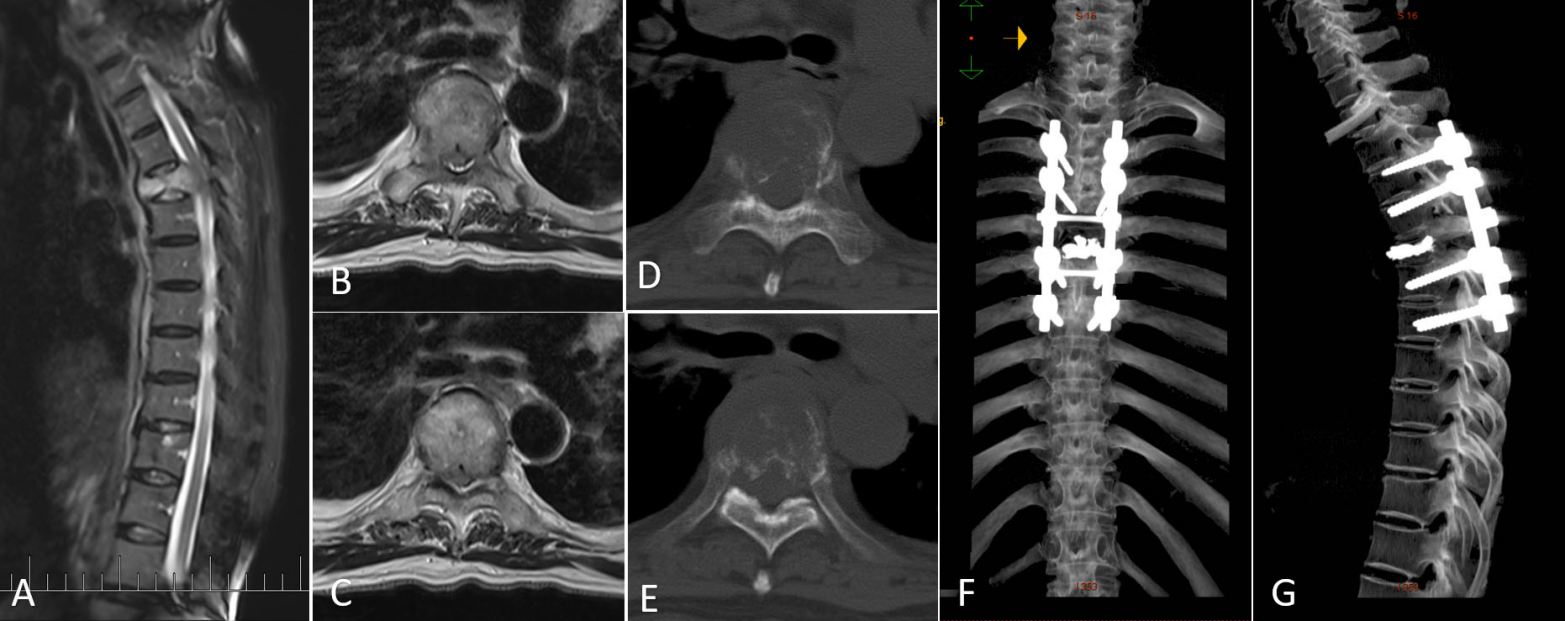
A representative case of metastatic spinal cord compression (MSCC) in lung cancer patients. **(A)** Sagittal MRI image showing MSCC at T5. **(B, C)** Transversal MRI images illustrating MSCC with epidural spinal cord compression (ESCC) score of 2. **(D, E)** Transversal CT images displaying osteolytic changes in T5. **(F, G)** Postoperative three-dimensional CT images.

Patients underwent a posterolateral approach for decompression of the vertebral canal and internal stabilization of the spine. The procedure involved the following steps: first, a fluoroscopy-guided incision was made along the posterior midline of the skin, followed by subcutaneous separation. The percutaneous pedicle screw fixation technique was employed to place screws through the fascia. To expose the posterior aspect of the spine, the surgeons made a small incision at the posterior center, cutting through the fascia and separating the muscles. The specific resection area depended on the extent of the tumor. After vertebrectomy, the vertebral body was reconstructed using techniques such as Kirschner wire cement, titanium mesh, or expandable interbody fusion cage. Finally, intra-fascial fixation rods were inserted and secured. The muscles and deep fascia were meticulously sutured using absorbable barbed thread, and the skin incision was closed using intradermal sutures. A case report illustrating the surgical site before and after surgery is depicted in **Figure 1**.

### Primary variable and definition

The primary variable of interest in this study was the HRQoL, which was assessed using the Functional Assessment of Cancer Therapy—General (FACT-G) questionnaire. The FACT-G is a reliable and valid instrument commonly used to evaluate HRQoL among lung cancer patients (16). Scores on the FACT-G range from 0 to 108, with higher scores indicating better overall HRQoL. In this study, poor HRQoL was defined as a FACT-G score of less than 60. Previous research has shown that FACT-G scores tend to peak at 3 months postoperatively and remain relatively stable during the follow-up period for patients with spinal metastasis (17). Consequently, patients were asked to self-report their current well-being as part of the FACT-G score collection 3 months after discharge from the hospital.

### Potential predictors and definitions

Based on a thorough literature review and data availability, several potential risk factors were included in the analysis. These factors encompassed patients’ demographics (e.g., age, sex, marital status, religious belief, education, smoking, and drinking status, time since cancer diagnosis, and uncompleted life goals), received treatments (e.g., surgery at the primary cancer site, radiotherapy, chemotherapy, and targeted therapy), tumor status (e.g., Eastern Cooperative Oncology Group (ECOG) score, visceral metastasis, and financial burden due to cancer treatments), number of comorbidities, and mental health status (e.g., anxiety and depression scores). Time since cancer diagnosis was defined as the interval between patients’ study participation date and the date of their cancer diagnosis. The number of comorbidities represented the cumulative sum of various medical conditions, including hypertension, diabetes, coronary heart disease, chronic liver disease, chronic renal disease, and asthma. Anxiety and depression scores were assessed using the Hospital Anxiety and Depression Scale (HADS), which is a widely used tool for evaluating mental health. All patients’ demographic and clinical characteristics were collected before surgery during their hospitalization.

### Construction of the nomogram

The least absolute shrinkage and selection operator (LASSO) regression analysis combined with 10-fold cross-validation was used to select variables for the development of the nomogram. One of the major advantages of LASSO regression is its ability to simultaneously perform variable selection and regularization (18, 19). This means that LASSO not only identifies the most relevant variables for prediction but also performs a degree of shrinkage, solving the problem of multi-collinearity in regression analysis and leading to improved model interpretability and reduced overfitting (18, 19).

### Validation of the nomogram

The overall performance of the nomogram was evaluated using the Brier index, which measures the discrepancy between the actual binary outcomes and the predicted risk probabilities. The Brier index ranges from 0 to 1, with values closer to 0 indicating a more accurate prediction model. A Brier index greater than 0.25 is typically considered indicative of a poor prediction model. In addition, the discriminative ability of the nomogram was assessed using the concordance index (C-index) and discrimination slope. The C-index, ranging from 0.5 to 1.0, measures how well the nomogram predicts the outcome, with values above 0.7 suggesting a favorable estimation. Furthermore, probability density curves were employed to visualize the predicted probabilities between patients with negative and positive events in both the updated and previous nomograms (20–23). Calibration plots and a goodness-of-fit test were used to evaluate the calibrating ability of the nomogram, with a p-value greater than 0.05 indicating good calibration. Sensitivity, specificity, accuracy, and the kappa index were calculated to further assess the performance of both the updated and previous nomograms.

### Clinical usefulness evaluation

The clinical benefits of the nomograms were compared using decision curve analysis (DCA), which plots net benefits against different threshold probabilities (24). In the decision curve, a reference line called the treat-for-all scheme represents the maximum clinical costs, while a reference line named the treat-for-none scheme indicates no clinical benefit. A decision curve positioned further away from these reference lines suggests a greater clinical value of the prediction model. Additionally, the net reclassification index (NRI) and integrated discrimination improvement (IDI) were employed to compare the nomogram with and without the number of comorbidities.

### Risk stratification of subgroups

To establish risk stratifications in this study, a cutoff point was determined based on an analysis of the threshold. The threshold was obtained through an area under the curve (AUC) analysis, where the predicted probability was plotted against the actual risk categories of “no” and “yes”. By plotting the predicted probabilities against the actual risk outcomes, researchers can identify the point where the model effectively distinguishes between the two categories. This point, known as the threshold, was subsequently used as the cutoff for risk stratification. Patients with a predicted probability of poor HRQoL below the threshold were classified into the low-risk group, while those above the threshold were classified into the high-risk group. The observed risk probabilities were calculated and compared between the low- and high-risk groups.

### Statistical analysis

Continuous variables were presented as mean ± standard deviation (SD) or median [interquartile range (IQR)], while categorical variables were described as proportions. The Wilcoxon two-sample and Kruskal–Wallis tests were used to compare differences between subgroups. All statistical analyses were performed using the R programming language and SAS 9.4 software, with a significance level set at p < 0.05 (two-tailed).

## Results

### Basic characteristics patients

A total of 119 lung cancer patients with MSCC were included in this study. The average age of the patients was 60.37 ± 10.49 years. The majority of patients were men (55.46%), were married (95.80%), had no religious beliefs (90.76%), and did not have a university education or above (76.48%). A large proportion of patients did not undergo surgery for their primary cancer site (76.47%), but most received radiotherapy (70.59%) and chemotherapy (68.07%). Many patients reported having unfinished wishes (76.47%). The comorbidity burden was relatively low, with only 15.12% of patients having two or more comorbidities. Demographic and clinical characteristics can be found in **Table 1**. The mean FACT-G score was 56.56 ± 17.88, indicating very poor HRQoL compared to the general population. Based on the definition in our study, 56.30% of the patients were classified as having positive events. In terms of mental health, the mean anxiety score was 9.02 ± 4.70, and the mean depression score was 8.48 ± 4.88, suggesting that these patients experienced severe mental distress. Furthermore, 89.91% of the patients reported a moderate-to-severe financial burden due to cancer treatments, indicating a heavy economic burden during the COVID-19 pandemic.

**Table 1:** Patient’s basic characteristics.

Characteristics	Patients (n = 119)
Age (mean ± SD, years)	60.37 ± 10.49
Sex	
Male	55.46%
Female	44.54%
Marital status	
Married	95.80%
Single	4.20%
Religious belief	
No	90.76%
Yes	9.24%
Education	
Primary education	37.82%
Senior high school	38.66%
University or above	23.53%
Smoking status	
No	52.94%
Quitting smoking	25.21%
Current smoking	21.85%
Drinking status	
No	70.59%
Quitting drinking	22.69%
Current drinking	6.72%
Time since knowing cancer diagnosis (months)	
<3	18.49%
≧3 and <6	14.29%
≧6 and <12	10.08%
≧12	57.14%
Visceral metastasis	
No	55.46%
Yes	44.54%
Surgery to the primary cancer site	
Open surgery	10.92%
Minimally invasive surgery	12.61%
None	76.47%
Radiotherapy	
No	29.41%
Yes	70.59%
Chemotherapy	
No	31.93%
Yes	68.07%
Financial burden due to cancer treatments	
None	1.68%
Mild	8.40%
Moderate	35.29%
Severe	54.62%
Having unfinished wishes	
No	23.53%
Yes	76.47%
ECOG scores	
0	5.04%
1	31.93%
2	31.93%
3	8.40%
4	22.69%
Tumor targeted therapy	
Yes	88.24%
No	11.76%
Number of comorbidities	
0	68.07%
1	16.81%
2	6.72%
≧3	8.40%
Anxiety score* (mean ± SD)	9.02 ± 4.70
Depression score* (mean ± SD)	8.48 ± 4.88
Relatively poor quality of life	
No	43.70%
Yes	56.30%
FACT-G score (mean ± SD)	56.56 ± 17.88
Physical (mean ± SD)	13.52 ± 6.76
Social/family (mean ± SD)	17.97 ± 5.92
Emotional (mean ± SD)	13.44 ± 5.14
Functional (mean ± SD)	11.64 ± 6.23

*indicates both scores were obtained based on the Hospital Anxiety and Depression Scale.

SD, standard deviation; ECOG, Eastern Cooperative Oncology Group; FACT-G, Functional Assessment of Cancer Therapy—General.

### Subgroup analysis of HRQoL

Subgroup analysis of HRQoL revealed significant differences in FACT-G scores based on smoking status (p = 0.03), drinking status (p = 0.03), visceral metastasis (p = 0.049), surgery to primary cancer site (p = 0.02), chemotherapy (p = 0.01), having unfinished wishes (p = 0.01), ECOG score (p < 0.0001), tumor-targeted therapy (p = 0.02), number of comorbidities (p = 0.003), anxiety status (p < 0.0001), and depression status (p < 0.0001) (**Table 2**). The same trend was observed in the subgroup analysis of the four FACT-G subscales. More detailed information can be found in **Table 3**.

**Table 2:** Subgroup analysis of quality of life among lung cancer patients with spine metastasis.

Characteristics	FACT-G score (median [IQR])	p
Age (years)		0.29^*^
<50	60.0 [45.0, 75.5]	
≥50 and <60	58.0 [41.0, 75.5]	
≥60 and <70	49.0 [41.0, 65.0]	
≥70	57.0 [48.0, 62.0]	
Sex		0.59
Male	57.0 [43.0, 65.0]	
Female	50.0 [41.0, 67.0]	
Marital status		0.56
Married	57.0 [41.0, 67.0]	
Single	50.0 [50.0, 63.0]	
Religious belief		0.08
No	57.0 [42.0, 67.0]	
Yes	43.0 [40.0, 60.0]	
Education		0.20^*^
Primary education	57.0 [42.0, 63.0]	
Senior high school	54.5 [41.0, 65.0]	
University or above	60.0 [45.5, 83.5]	
Smoking status		0.03^*^
No	57.0 [41.0, 75.0]	
Quitting smoking	58.5 [56.0, 65.0]	
Current smoking	49.0 [37.0, 59.0]	
Drinking status		0.03^*^
No	57.0 [41.0, 67.0]	
Quitting drinking	57.0 [51.0, 65.0]	
Current smoking	35.5 [28.0, 47.5]	
Time since knowing cancer diagnosis (months)		0.98^*^
<3	57.0 [42.0, 66.0]	
≧3 and <6	60.0 [45.0, 65.0]	
≧6 and <12	57.0 [46.0, 77.0]	
≧12	50.5 [41.0, 67.0]	
Visceral metastasis		0.049
No	60.0 [49.0, 75.0]	
Yes	53.0 [40.0, 62.0]	
Surgery to primary cancer site		0.02^*^
Open surgery	66.0 [56.0, 81.0]	
Minimally invasive surgery	39.0 [29.0, 60.0]	
None	57.0 [43.0, 66.0]	
Radiotherapy		0.15
No	60.0 [50.0, 66.0]	
Yes	54.5 [41.0, 66.5]	
Chemotherapy		0.01
No	62.0 [53.0, 67.0]	
Yes	51.0 [40.0, 65.0]	
Financial burden due to cancer treatments		0.31^*^
None	42.0 [42.0, 42.0]	
Mild	61.5 [53.0, 88.0]	
Moderate	56.5 [40.0, 65.0]	
Severe	57.0 [41.0, 67.0]	
Having unfinished wishes		0.01
No	61.5 [45.0, 85.5]	
Yes	56.0 [40.0, 65.0]	
ECOG scores		<0.0001^*^
0	71.0 [27.0, 88.0]	
1	70.0 [57.0, 81.0]	
2	57.0 [51.0, 63.0]	
3	49.0 [37.0, 50.0]	
4	41.0 [35.0, 45.0]	
Tumor targeted therapy		0.02
Yes	57.0 [43.0, 67.0]	
No	50.0 [30.0, 56.0]	
Number of comorbidities		0.003^*^
0	60.0 [45.0, 67.0]	
1	49.0 [40.0, 62.0]	
2	53.0 [50.0, 86.0]	
≧3	34.5 [29.0, 49.0]	
Anxiety		<0.0001^*^
No	65.0 [57.0, 79.5]	
Skeptical	57.0 [56.0, 63.0]	
Yes	41.0 [37.0, 50.0]	
Depression		<0.0001^*^
No	63.0 [56.0, 76.0]	
Skeptical	53.5 [41.0, 66.0]	
Yes	41.0 [35.0, 50.0]	
Relatively poor quality of life		<0.0001
No	67.0 [63.0, 81.0]	
Yes	43.0 [37.0, 52.0]	

^*^indicates the p-values were obtained from the Kruskal–Wallis test, and others were obtained from the Wilcoxon two-sample test.

IQR, interquartile range; ECOG, Eastern Cooperative Oncology Group; FACT-G, Functional Assessment of Cancer Therapy—General.

**Table 3:** Subgroup analysis of the four FACT-G subscales among lung cancer patients with spine metastasis.

Characteristics	FACT-G subscales (median [IQR])			
	Physical well-being	Social/family well-being	Emotional well-being	Functional well-being
Age (years)				
<50	18.0 [6.5, 20.5]	18.0 [15.0, 19.5]	16.0 [11.0, 18.0]	13.0 [9.5, 21.0]
≥50 and <60	15.0 [9.0, 21.0]	22.0 [15.0, 24.0]	16.0 [12.5, 18.0]	11.5 [6.0, 18.0]
≥60 and <70	12.5 [9.0, 16.0]	18.0 [15.0, 22.0]	14.0 [9.0, 15.0]	10.5 [5.0, 13.0]
≥70	15.0 [7.0, 19.0]	17.0 [15.0, 21.0]	13.0 [10.0, 15.0]	12.0 [10.0, 14.0]
Sex				
Male	15.0 [8.0, 19.0]	18.0 [14.0, 21.0]	14.0 [12.0, 17.0]	11.0 [8.0, 15.0]
Female	13.0 [6.0, 19.0]	20.0 [16.0, 23.0]	14.0 [9.0, 18.0]	11.0 [6.0, 15.0]
Marital status				
Married	14.0 [8.0, 19.0]	18.0 [15.0, 22.0]	14.0 [9.0, 18.0]	11.0 [7.0, 15.0]
Single	12.0 [12.0, 16.0]	6.0 [6.0, 23.0]	14.0 [14.0, 17.0]	11.0 [11.0, 14.0]
Religious belief				
No	15.0 [9.0, 19.0]	18.0 [15.0, 22.0]	14.0 [11.5, 17.0]	11.0 [7.0, 15.0]
Yes	6.0 [5.0, 14.0] ^**^	20.0 [17.0, 24.0]	9.0 [2.0, 18.0] ^*^	11.0 [4.0, 17.0]
Education				
Primary education	15.0 [9.0, 19.0]	18.0 [15.0, 21.0]	14.0 [12.0, 17.0]	11.0 [7.0, 13.0]
Senior high school	11.5 [6.0, 17.0] ^*^	19.0 [15.0, 22.0]	14.0 [8.0, 17.0]	11.5 [7.0, 14.0]
University or above	17.0 [12.0, 21.0]	18.5 [14.5, 23.5]	16.0 [13.0, 18.0]	13.5 [9.0, 19.5]
Smoking status				
No	14.0 [7.0, 20.0]	21.0 [16.0, 23.0]	14.0 [8.0, 18.0]	11.0 [7.0, 15.0]
Quitting smoking	16.0 [8.0, 19.0]	18.0 [16.0, 21.0]	14.0 [12.0, 16.0]	12.0 [11.0, 15.0]
Current smoking	13.0 [9.0, 16.0]	14.5 [10.0, 20.0] ^***^	14.0 [9.0, 17.0]	8.0 [5.0, 10.0] ^*^
Drinking status				
No	15.0 [8.0, 19.5]	18.0 [16.0, 22.0]	14.0 [9.0, 18.0]	11.0 [7.0, 14.0]
Quitting drinking	14.0 [11.0, 18.0]	18.0 [12.0, 23.0]	15.0 [12.0, 17.0]	13.0 [9.0, 19.0]
Current smoking	8.0 [6.0, 14.0]	10.5 [8.0, 20.0]	9.5 [6.5, 13.0]	6.0 [4.5, 7.5] ^**^
Time since knowing cancer diagnosis (months)				
<3	16.0 [7.0, 19.0]	17.0 [15.0, 20.0]	13.0 [11.0, 14.0]	11.5 [7.0, 14.0]
≧3 and <6	18.0 [8.0, 19.0]	18.0 [15.0, 22.0]	13.0 [11.0, 16.0]	11.0 [4.0, 14.0]
≧6 and <12	14.5 [10.0, 18.5]	17.0 [16.0, 21.0]	15.0 [11.5, 18.0]	11.5 [6.0, 21.0]
≧12	13.0 [8.0, 19.0]	20.5 [14.5, 23.0]	14.5 [8.5, 18.0]	11.0 [7.0, 15.0]
Visceral metastasis				
No	16.0 [11.0, 21.0]	18.0 [13.0, 22.0]	14.0 [12.0, 18.0]	12.0 [8.0, 15.0]
Yes	11.0 [6.0, 17.0] ^***^	20.0 [16.0, 22.0]	14.0 [9.0, 17.0]	10.0 [6.0, 13.0] ^*^
Surgery to primary cancer site				
Open surgery	14.0 [6.0, 21.0]	21.0 [18.0, 22.0]	17.0 [11.0, 20.0]	21.0 [11.0, 22.0]
Minimally invasive surgery	9.0 [7.0, 15.0]	12.0 [7.0, 22.0]	11.0 [6.0, 14.0]	7.0 [6.0, 17.0]
None	15.0 [8.0, 19.0]	18.0 [15.0, 22.0] ^*^	14.0 [9.0, 18.0] ^*^	11.0 [7.0, 14.0] ^*^
Radiotherapy				
No	17.0 [13.0, 19.0]	16.0 [13.0, 21.0]	14.0 [13.0, 17.0]	11.0 [10.0, 17.0]
Yes	12.0 [6.5, 19.0] ^*^	20.0 [16.0, 22.0]	14.0 [9.0, 18.0]	11.0 [6.5, 14.0]
Chemotherapy				
No	18.0 [12.0, 20.0]	18.5 [15.0, 22.0]	14.0 [12.0, 19.0]	12.0 [8.0, 14.0]
Yes	13.0 [7.0, 17.0] ^**^	18.0 [15.0, 22.0]	14.0 [9.0, 17.0]	11.0 [7.0, 17.0]
Financial burden due to cancer treatments				
None	5.0 [5.0, 5.0]	20.0 [20.0, 20.0]	12.0 [12.0, 12.0]	5.0 [5.0, 5.0]
Mild	17.5 [10.0, 21.0]	19.0 [10.0, 24.0]	17.5 [13.0, 20.0]	12.5 [8.0, 23.0]
Moderate	13.0 [11.0, 17.0]	18.0 [14.0, 21.0]	14.0 [11.0, 16.0]	11.0 [7.0, 17.0]
Severe	15.0 [7.0, 19.0]	19.0 [15.0, 23.0]	14.0 [8.0, 18.0]	11.0 [7.0, 14.0]
Having unfinished wishes				
No	18.0 [10.0, 22.0]	20.0 [17.0, 22.0]	18.0 [13.0, 19.5]	11.0 [7.5, 20.5]
Yes	13.0 [7.0, 19.0]	18.0 [15.0, 22.0]	14.0 [9.0, 16.0] ^****^	11.0 [7.0, 14.0]
ECOG scores				
0	14.0 [7.0, 23.0]	20.5 [7.0, 25.0]	16.0 [6.0, 18.0]	17.0 [7.0, 23.0]
1	20.0 [16.0, 21.0]	19.5 [16.0, 23.0]	15.5 [13.0, 18.0]	15.0 [11.0, 20.0]
2	13.5 [11.0, 18.0]	16.0 [13.0, 21.0]	15.0 [13.0, 18.0]	10.5 [7.0, 13.0]
3	11.0 [5.0, 11.0]	19.0 [14.0, 21.0]	12.0 [5.0, 14.0]	8.0 [6.0, 11.0]
4	5.0 [1.0, 7.0] ^****^	20.0 [18.0, 24.0] ^*^	9.0 [6.0, 12.0] ^****^	4.0 [3.0, 13.0] ^****^
Tumor targeted therapy				
Yes	15.0 [7.0, 19.0]	20.0 [16.0, 22.0]	14.0 [9.0, 18.0]	12.0 [7.0, 15.0]
No	11.0 [9.0, 14.0]	14.0 [5.0, 16.0] ^****^	14.0 [13.0, 15.0]	9.0 [7.0, 11.0]
Number of comorbidities				
0	15.0 [8.0, 20.0]	18.0 [15.0, 22.0]	15.0 [11.0, 18.0]	12.0 [8.0, 17.0]
1	12.5 [6.0, 19.0]	20.5 [17.0, 23.0]	12.0 [9.0, 14.0]	8.0 [4.0, 12.0]
2	18.0 [11.0, 23.0]	16.5 [12.0, 23.0]	17 [14, 19]	13.0 [9.5, 19.0]
≧3	9.0 [9.0, 11.0] ^*^	12.0 [4.0, 18.0] ^*^	12.0 [7.0, 12.0] ^**^	7.0 [4.0, 11.0] ^**^
Anxiety				
No	19.0 [13.5, 21.0]	19.0 [15.0, 22.0]	17.0 [14.5, 19.0]	13.0 [9.5, 17.0]
Skeptical	15.0 [12.0, 15.0]	16.0 [15.0, 21.0]	14.0 [12.0, 15.0]	12.0 [11.0, 17.0]
Yes	7.5 [5.0, 11.0] ^****^	18.0 [14.0, 22.0]	9.0 [6.0, 14.0] ^****^	6.5 [4.0, 11.0] ^****^
Depression				
No	19.0 [14.0, 20.0]	20.0 [15.0, 23.0]	15.0 [14.0, 18.0]	13.0 [10.0, 18.0]
Skeptical	12.5 [8.0, 16.0]	17.5 [12.0, 21.0]	14.0 [9.0, 18.0]	12.0 [8.0, 17.0]
Yes	9.0 [5.0, 13.0] ^****^	17.0 [14.0, 21.0]	10.5 [6.0, 14.0] ^****^	5.5 [4.0, 11.0] ^****^
Relatively poor quality of life				
No	19.0 [16.0, 21.0]	21.0 [17.5, 23.0]	17.5 [15.0, 19.0]	15.5 [13.0, 19.5]
Yes	9.0 [6.0, 14.0] ^****^	16.0 [13.0, 20.0] ^****^	11.0 [7.0, 14.0] ^****^	8.0 [4.0, 11.0] ^****^

*indicates p < 0.05; **indicates p < 0.01; ***indicates p < 0.001; ****indicates p < 0.0001.

FACT-G, Functional Assessment of Cancer Therapy—General; IQR, interquartile range; ECOG, Eastern Cooperative Oncology Group.

### Nomogram variable screening

The LASSO method combined with 10-fold cross-validation identified three variables for the development of the nomogram: ECOG score, targeted therapy, and anxiety score (**Supplementary Figure 2**). Moreover, this study found that the scores of the four FACT-G subscales were all significantly different distributed in the variable of the number of comorbidities (**Table 3**), indicating this variable might also play an important role in affecting HRQoL among those patients. Furthermore, after a thorough review of the literature, this variable was also found to be significantly associated with HRQoL in the previous studies (25, 26). Thus, the variable was also included in the nomogram. As a result, four variables were included in the final nomogram. Notably, the coefficients between the four variables were all below 0.63 based on Spearman’s rank correlation analysis, indicating that no serious collinearity existed between the four variables.

### Construction of the nomogram

**Figure 2:**
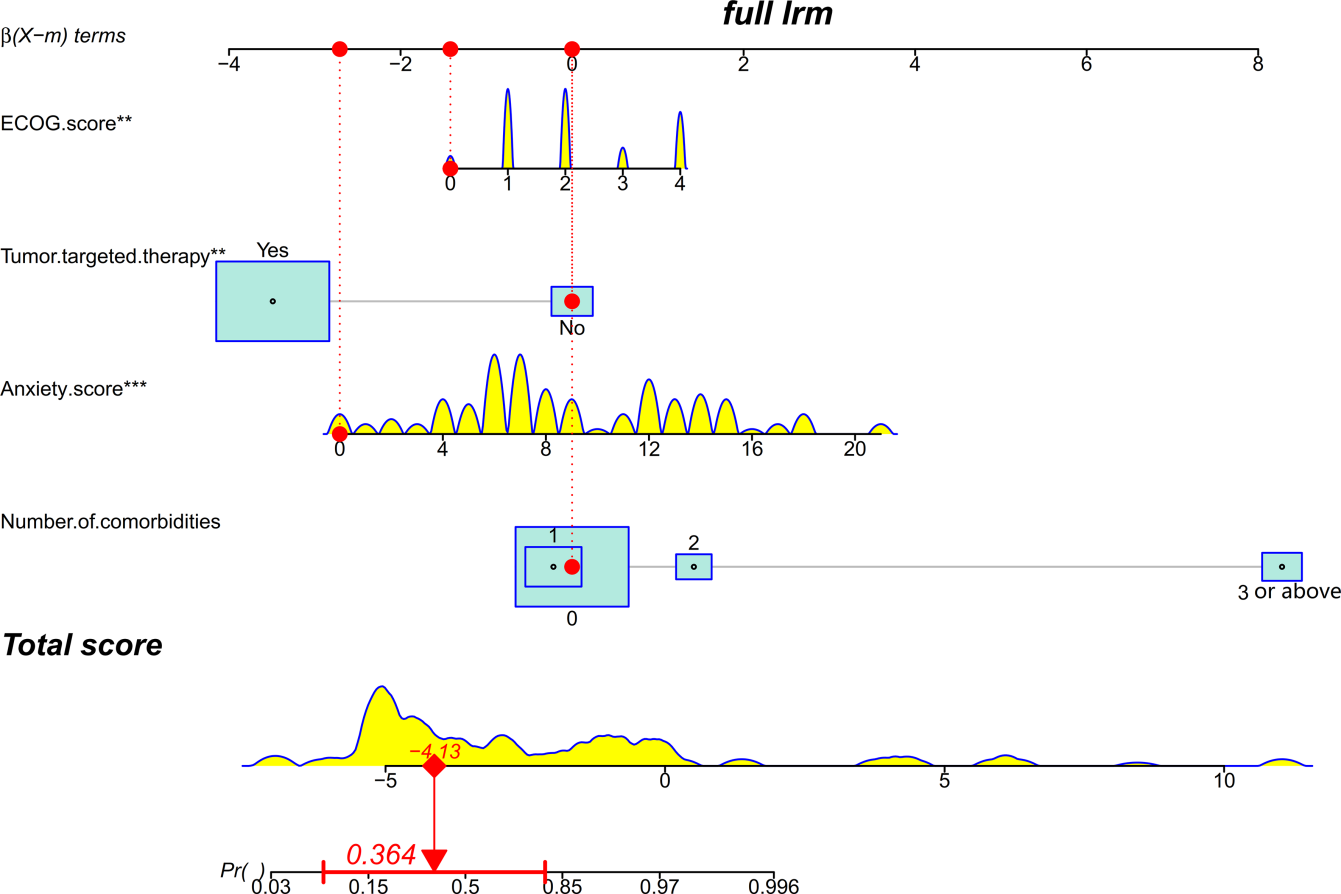
A nomogram for predicting and stratifying health-related quality of life (HRQoL) in lung cancer patients with metastatic spinal cord compression (MSCC). The density plot represents the distribution of continuous variables including the Eastern Cooperative Oncology Group (ECOG) score, anxiety score, and total score, while the size of the box represents the distribution of proportional variables including targeted therapy and number of comorbidities. In this example, the patient had an ECOG score of 0, did not receive targeted therapy, had an anxiety score of 0, and had three comorbidities. The red dots on each variable axis represent the patient’s actual condition, and red lines are drawn upward to the corresponding points on the points axis. The total points are obtained by adding up the points from all four variables, and the sum value (−0.638) is located on the total score axis. A line is drawn downward to the risk probability axis to determine the predicted probability (36.40%) of poor HRQoL. The horizontal red line on the risk probability axis indicates the 95% confidence interval of the predicted probability.

The final nomogram was constructed using the four variables based on the logistic regression model (**Figure 2**). The nomogram showed that a higher ECOG score, targeted therapy, a higher anxiety score, and a large number of comorbidities were associated with a high-risk probability of poor HRQoL. The variables were ranked by the SD, with the top three variables being the ECOG score, targeted therapy, and anxiety score. An example of using the nomogram to predict the probability of poor HRQoL in a given patient was also provided.

### Validation of the nomogram

**Figure 3:**
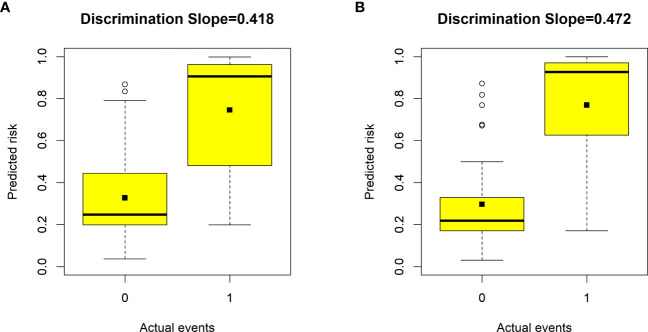
Box plots showing the discriminative slope of predicted risk probabilities between patients with and without poor health-related quality of life (HRQoL). **(A)** Discrimination slope for the previous nomogram (excluding the number of comorbidities). **(B)** Discrimination slope for the updated nomogram (including the number of comorbidities). The discrimination slope represents the difference in mean predicted probability between patients with and without poor HRQoL (solid dots indicate means). 0 indicates a negative event, and 1 indicates a positive event.

**Figure 4:**
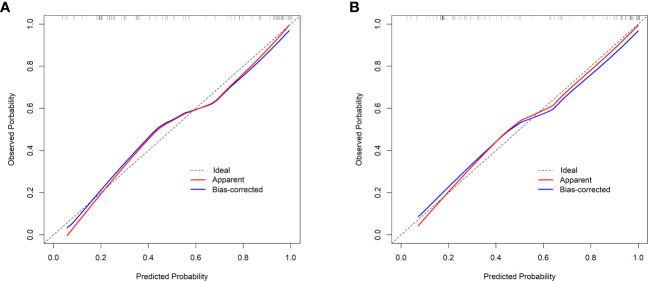
Calibration curves. **(A)** The previous nomogram (excluding the number of comorbidities). **(B)** The updated nomogram (including the number of comorbidities). The calibration curves plot the predicted probability against the observed probability. The ideal reference line is shown as a dotted line, indicating a perfect match between the predicted and observed probabilities. The red solid line represents the apparent calibration of the nomogram, and the blue solid line represents the bias-corrected calibration of the nomogram.

Validation of the nomogram showed that the addition of the number of comorbidities improved the overall performance of the nomogram, as indicated by a lower Brier index (0.13 *vs.* 0.15) and a higher C-index value (0.87 ± 0.10 *vs.* 0.85 ± 0.11) (**Table 4**). A similar trend was also observed regarding discrimination slope (0.42 *vs.* 0.47, **Figure 3**), sensitivity (77.67% ± 17.94% *vs.* 80.23% ± 18.26%), and specificity (71.89% ± 16.97% *vs.* 76.41 ± 15.67%). The probability density curves were plotted for the previous nomogram (**Supplementary Figure 3A**) and the updated nomogram (**Supplementary Figure 3B**). In patients with positive events, the peak of the density curve was located at a high level of predicted probability of poor HRQoL, while in patients with negative events, the peak moved to a very low level of predicted probability. This indicates that the nomogram effectively differentiated patients with different HRQoL outcomes. Furthermore, the updated nomogram showed a larger gap between the density peaks of positive and negative events, indicating a stronger discriminatory ability. The calibration plot of the previous nomogram (**Figure 4A**) demonstrated good consistencies between the predicted and observed probability. The curve was generally close to the ideal reference line, indicating that the predicted probabilities aligned well with the actual probabilities of poor HRQoL. However, in the updated nomogram (**Figure 4B**), the curve was even closer to the ideal reference line, showcasing higher consistencies between the predicted and observed probabilities. This also suggests that the updated nomogram improved the accuracy of predicting HRQoL outcomes and provided a more reliable estimation of the true probabilities. The p-values of the goodness-of-fit test for both nomograms were above 0.05, suggesting significant consistencies between the predicted and observed probabilities in both models.

**Table 4:** Prediction performance of the nomogram to predict poor quality of life among lung cancer patients with spine metastasis.

Parameters	Previous nomogram	Updated nomogram
Overall performance		
Brier	0.15	0.13
Discriminative ability		
C-index^*^	0.85 ± 0.11	0.87 ± 0.10
Discrimination slope	0.42	0.47
Sensitivity^*^	77.67% ± 17.94%	80.23% ± 18.26%
Specificity^*^	71.89% ± 16.97%	76.41% ± 15.67%
Calibrating ability		
p-Value^#^	0.28	0.13
NRI (categorical) ^*^ [95% CI]	0.113 [0.002–0.224], p-value: 0.046	
NRI (continuous) ^*^ [95% CI]	0.404 [0.074–0.734], p-value: 0.016	
IDI^*^ [95% CI]	0.035 [0.004–0.066], p-value: 0.027	

^*^indicates that the values were obtained using the bootstrapping method with 1,000 iterations of procedures; ^#^indicates that the p-value was obtained from the goodness-of-fit test.

C-index, concordance index; NRI, net reclassification index; CI, confidence interval; IDI, integrated discrimination improvement.

### Clinical value of the updated nomogram compared with the previous nomogram

**Figure 5:**
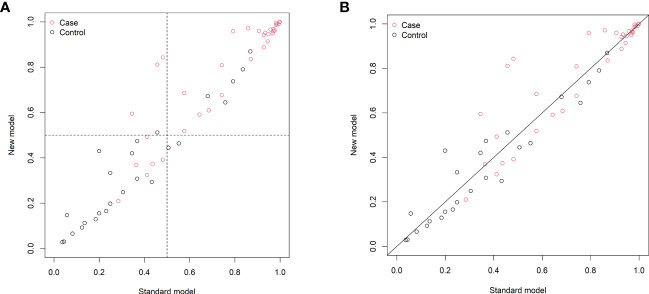
Reclassification of patients in the previous (standard model) and updated (new model) nomograms. **(A)** The categorical net reclassification index (0.113, 95% CI: 0.002–0.224, p = 0.046). **(B)** The continuous net reclassification index (0.404, 95% CI: 0.074–0.734, p = 0.016).

**Figure 6:**
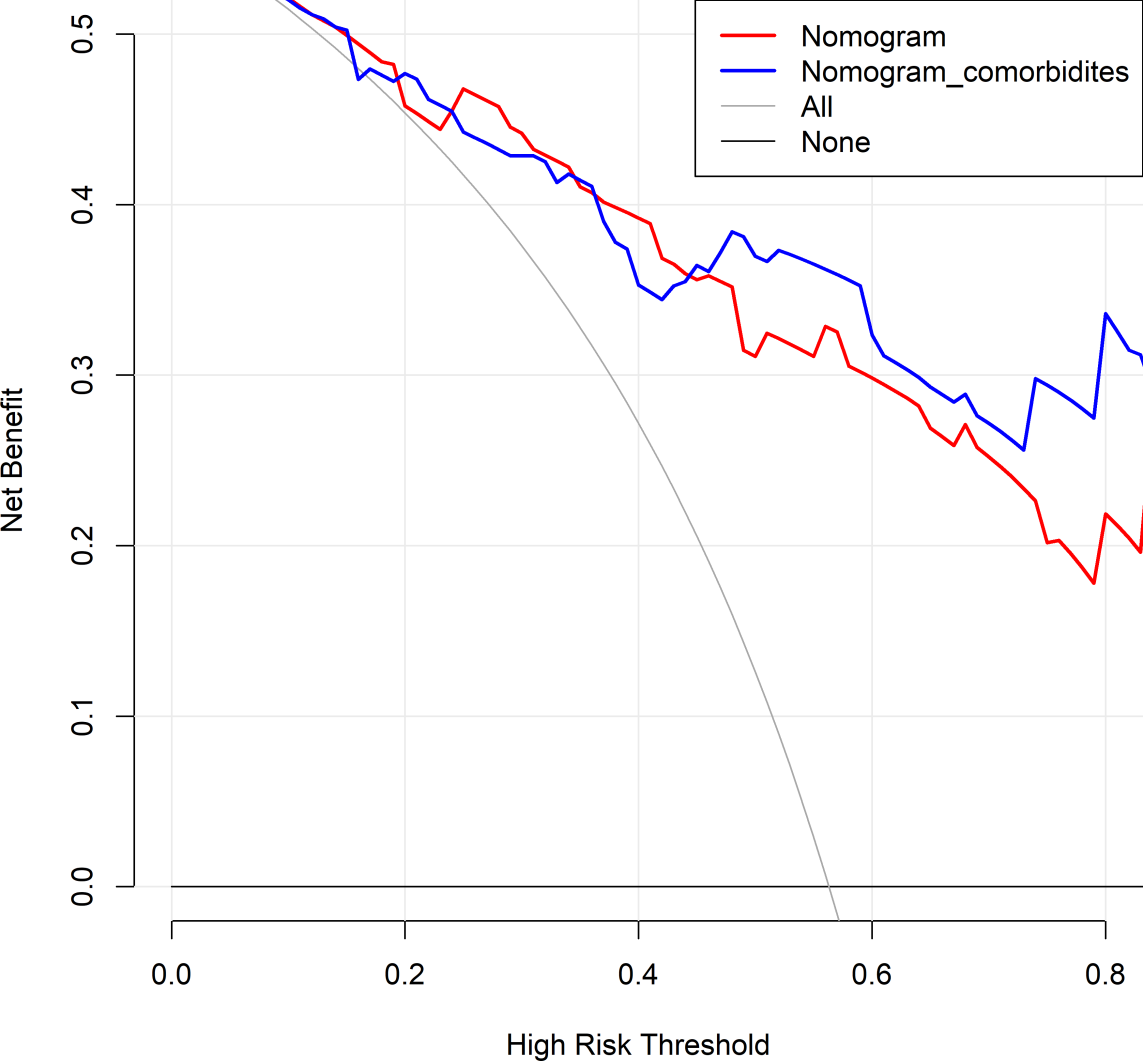
Decision curve analysis comparing the previous and updated nomograms for predicting health-related quality of life (HRQoL) in lung cancer patients with metastatic spinal cord compression (MSCC). The horizontal black line represents the treated-for-none scheme, and the solid gray line represents the treated-for-all scheme. The red line represents the previous nomogram, and the blue line represents the updated nomogram.

The changes in NRI and IDI were used to compare the accuracy between the previous nomogram and the updated nomogram. The categorical NRI was 0.113 (95% CI: 0.002–0.224, p = 0.046, **Figure 5A**), and the continuous NRI was 0.404 (95% CI: 0.074–0.734, p = 0.016, **Figure 5B**). The IDI was 0.035 (95% CI: 0.004–0.066, p = 0.027). These results indicated that the updated nomogram predicted HRQoL with greater accuracy than the previous nomogram. The clinical benefits of the updated nomogram were compared with those of the previous nomogram. DCA curves showed that the updated nomogram could better predict HRQoL, as it added more net benefits compared with the previous nomogram, especially for high threshold probabilities (**Figure 6**).

### Risk stratification based on the nomogram

Based on the nomogram, risk stratification was performed using a cutoff point of 50.00% for the predicted probability of poor HRQoL. Patients with a predicted probability of poor HRQoL of less than 50.00% were classified into the low-risk group, and those with 50.00% or above were classified into the high-risk group. In the previous nomogram, the corresponding observed risk probabilities in the low-risk and high-risk groups were 31.67% and 81.36%, respectively (p < 0.001, **Table 5**). In the updated nomogram, the observed risk probability decreased to 23.73% in the low-risk group and increased to 88.33% in the high-risk group (**Table 6**), indicating that the updated nomogram had a better ability to recognize patients in the high- or low-risk groups. Moreover, the observed risk probabilities were more similar to the predicted risk probabilities among both risk groups based on the updated nomogram.

**Table 5:** Risk stratification of quality of life among lung cancer patients with spine metastasis based on the nomogram without comorbidities.

Risk groups	Risk probability		p^*^
	Predicted	Observed (n = 119)	
Low-risk group (<50.00%)	26.85% ± 12.18%	31.67% (19/60)	<0.001
High-risk group (≥50.00%)	86.26% ± 14.05%	81.36% (48/59)

^*^indicates p was obtained from the chi-square test for a comparison of observed probability between the low- and high-risk groups.

**Table 6:** Risk stratification of quality of life among lung cancer patients with spine metastasis based on the nomogram with comorbidities.

Risk groups	Risk probability		p^*^
	Predicted	Observed (n = 119)	
Low-risk group (<50.00%)	24.01% ± 11.72%	23.73% (14/59)	<0.001
High-risk group (≥50.00%)	88.06% ± 13.49%	88.33% (53/60)

^*^indicates p was obtained from the chi-square test for a comparison of observed probability between the low- and high-risk groups.

## Discussion

Spinal metastatic lung cancer is a prevalent tumor, but there is limited clinical evidence available regarding the prediction of HRQoL in this population. Assessing HRQoL can guide healthcare providers in meeting the supportive care needs of patients with advanced-stage cancer. To address this gap, our study aimed to develop a nomogram for predicting HRQoL in patients with lung cancer and spinal metastasis. The nomogram included four variables: the ECOG score, targeted therapy, anxiety score, and number of comorbidities. The nomogram provides a visually intuitive representation of the prediction model, allowing healthcare professionals to estimate individual patient outcomes based on the relevant predictors included in the model. It simplifies the process of calculating predicted probabilities and facilitates the interpretation of the model’s performance.

To begin with, the present study found that metastatic lung cancer patients suffered from very poor HRQoL, as shown by the results that the mean FACT-G score was only 56.56 ± 17.88, which was significantly lower than that among the general population and even other advanced cancer patients (the mean FACT-G score was approximately 70.00 (27)). The reasons might be as follows: first, the present study was designed especially for lung cancer patients with metastatic spinal disease and spine metastasis, which could result in severe functional disability, and this was confirmed by our data based on ECOG score: only 5.04% had normal daily activity, and up to 22.69% of patients totally lost their self-care ability. Thus, the symptom burden of those patients might be more serious than general advanced cancer patients. Second, the present study was conducted during the COVID-19 pandemic, and the implementation of lockdown measures and self-isolation protocols aimed to control the spread of the virus, which possibly resulted in significant emotional issues and impaired HRQoL among patients (9). The restrictions on mobility, reduced social interactions, and limited access to healthcare services during lockdowns might have contributed to increased feelings of loneliness, anxiety, and depression (9), ultimately impacting the HRQoL outcomes observed in the study. Third, new strategies of adjuvant therapy, such as target therapy, immune therapy, and stereotactic radiotherapy, have been rapidly developed in recent years (16). High medical costs led to heavy economic burdens on these patients and their family members. In the present study, nearly 90% of patients reported moderate-to-severe financial burden due to cancer treatments, which also could partly explicate the poor HRQoL among our cohort. The unaffordability of medical expenses and limited access to necessary healthcare resources due to financial constraints could have further impaired their HRQoL. Therefore, recognizing the potential impact of the above factors on patient well-being is crucial in understanding the context in which the study was conducted and the generalizability of the findings to other settings or time periods.

According to previous studies, there were models to predict quality-of-life outcomes among patients with pancreatic cancer (28), cervical cancer survivors (29), colorectal cancer survivors (30, 31), patients who underwent colorectal cancer surgery (32), and patients who underwent prostate radiation therapy (33). In addition, a prospective study conducted by Nater et al. (34) developed a model to predict quality-of-life outcomes 3 months after surgery among 258 patients with MSCC due to various cancer sources. The final model consisted of the Karnofsky performance status, living in North America, SF-36 physical component score, and SF-36 mental component score, for a total number of predictor degrees of freedom of 4. The average c-statistic for the 10 imputed datasets was 0.72, and the corrected optimism was 0.74. Notably, in our study, the nomogram was initially designed for patients with metastatic spinal cord compression particularly secondary to lung cancer. In addition, the C-index was 0.87, indicating very favorable prediction performance. In our nomogram, we also included four model predictors, because a higher ECOG score, no targeted therapy, a higher anxiety score, and a larger number of comorbidities were associated with poorer HRQoL. Previous studies had already demonstrated that ECOG scores were closely relevant to HRQoL (35–38). For example, Daly et al. (35) showed that an ECOG score of 3 or 4 was independently related to poorer HRQoL among incurable cancer patients, and the odd ratio could be up to 14.33, indicating the importance of the variable. Engelhardt et al. (36) also confirmed that the ECOG score was the strongest determinant for HRQoL among multiple myeloma patients in Germany. In the present study, the ECOG score ranked first in the nomogram. Other studies also demonstrated the association between ECOG score and HRQoL among advanced cancer patients (37, 38). In the present study, targeted therapy was found to be a positive contributor to improving HRQoL among lung cancer patients. This finding was consistent with other previous studies (39, 40). For instance, Petrillo et al. (39) concluded that lung cancer patients with targeted therapy experienced improved HRQoL and symptoms in a secondary analysis of a randomized trial, and it is possibly due to the benefits of targeted therapy in controlling the progress of the disease. A review also summarized that targeted therapy brought great benefits to survival and quality of life in the context of advanced disease among lung cancer patients (40). Expectedly, anxiety was a detrimental factor to HRQoL. Morrison et al. (8) demonstrated that greater severity of emotional problems was strongly associated with lower quality of life in lung cancer patients. Polanski et al. (41) also showed that the intensity of anxiety was related to HRQoL in lung cancer patients. A similar result was also shown by Khue et al. (42). Previous studies had demonstrated that comorbidity was an influencing factor of HRQoL among cancer survivors (25). According to a systematic review, the presence of comorbidities was also found to be associated with poorer HRQoL (26). Moreover, in the present study, the scores of the four FACT-G subscales were all significantly different distributed in the number of comorbidities, suggesting the variable might also play an important role in affecting HRQoL. When incorporating the variable into the nomogram, the predictive performance of the nomogram was improved in terms of calibration and discrimination.

This study divided patients into low- and high-risk groups according to the nomogram. In the final nomogram, the observed risk probability was 23.73% in the low-risk group and 88.33% in the high-risk group, indicating that the patients in the high-risk group were nearly four times more likely to develop poor HRQoL than patients in the low-risk group. The nomogram elucidated potential clinical value in practice. The bootstrapping method was used to calculate C-index, sensitivity, and specificity, and plot calibration curves. NRI and IDI were also calculated using the bootstrapping method. Overall, the final nomogram could be a useful tool to assess HRQoL among lung cancer patients with metastatic spinal disease. The implementation of this nomogram in routine clinical practice can bring several benefits. First, it assists clinicians in making informed treatment decisions by providing personalized predictions of patients’ quality-of-life outcomes. By incorporating relevant clinical variables into the nomogram, clinicians can assess the potential impact of different treatment options on the quality of life of lung cancer patients with MSCC. The nomogram also enhances patient–physician communication. The visual representation of the model facilitates patient understanding of the predicted outcomes and enables them to actively participate in shared decision-making. Having access to personalized predictions can empower patients to consider their treatment options more comprehensively and align them with their individual preferences and goals. Nonetheless, the integration of the nomogram into routine clinical practice might face certain challenges and barriers. First, adequate training and education of healthcare professionals are necessary to ensure the proper use and interpretation of the nomogram. Clinicians need to be familiar with the predictors included in the model and understand how to input patient-specific information to derive individualized predictions accurately. Additionally, the nomogram may require periodic updating to ensure its performance and account for changes in treatment protocols or patient populations. Regular validation and recalibration of the nomogram using new data can help maintain its predictive accuracy over time. Finally, there may be initial resistance or skepticism from some healthcare professionals regarding the adoption of the nomogram as an additional tool in clinical decision-making. Addressing concerns, providing evidence of its clinical utility, and promoting its benefits through educational initiatives and collaborative efforts can help overcome these barriers and facilitate its integration into routine clinical practice.

## Limitations

Despite the valuable findings of this study, several limitations need to be acknowledged. First, the limited sample size restrained the ability to perform data splitting for internal validation, meaning that the model was established and internally validated using the entire dataset. To enhance the robustness of the findings, future work should focus on collecting a larger number of clinical samples to ensure better representation and generalizability of the predictive model. Additionally, conducting a multicenter clinical validation is crucial to assess the external replicability of the developed nomogram. Although the current validation of the prediction model demonstrated favorable performance, it is essential to test its effectiveness and reliability across different healthcare settings. Notably, this study employed self-reported measures, namely, the FACT-G and HADS scales, to evaluate HRQoL and mental health. Consequently, it is challenging to completely eliminate recall bias in this context. Furthermore, certain variables, such as surgical margins and lymphatic metastasis, were not included in the study’s evaluation. Incorporating these additional variables into the nomogram may enhance its predictive performance, providing a more comprehensive and accurate assessment of individual risk. Lastly, future research efforts should explore more advanced prediction model methods (43). Implementing these advanced techniques can help enhance the prediction performance of the nomogram and provide deeper insights into the clinical implications of the model, improving its interpretability and utility in real-world scenarios.

## Conclusions

A prediction nomogram is developed to guide clinicians in evaluating HRQoL among lung cancer patients with MSCC. This user-friendly tool encompasses a multitude of easily obtainable variables, enabling clinicians to make accurate predictions regarding postoperative HRQoL. Moreover, the nomogram possesses the remarkable ability to provide personalized forecasts, effectively identifying and stratifying patients who are likely to experience suboptimal HRQoL and prompting timely interventions or the selection of appropriate patients for treatment. Nonetheless, further multicenter clinical validation is imperatively needed to corroborate its reliability and effectiveness.

## Data availability statement

The data are available under reasonable request to the corresponding authors. Requests to access these datasets should be directed to YL, 632763246@qq.com.

## Ethics statement

This study was approved by the Ethics Committee of the Fourth Medical Center of Chinese PLA General Hospital. Informed written consent was obtained from all patients and all data were anonymously collected. This study complied with the Declaration of Helsinki. The studies were conducted in accordance with local legislation and institutional requirements. The participants provided their written informed consent to participate in this study.

## Author contributions

All authors contributed to the study design, conducted the data collection and analyses, and drafted the paper. All authors contributed to the article and approved the submitted version.

## Supplementary material

Supplementary Figure 1 Flowchart depicting the patient selection process.

Supplementary Figure 2 Identification of variables using least absolute shrinkage and selection operator (LASSO) regression analysis combined with ten-fold cross-validation. **(A)** Coefficients of all variables based on the variable-screening method. **(B)** Selection of the appropriate log(λ) parameter based on the variable-screening method. The left dotted vertical line indicates the optimal value according to the minimum criteria, and the right dotted vertical line indicates one standard error of the minimum criteria.

Supplementary Figure 3 Risk probability density curve for patients with and without poor HRQoL. **(A)** The previous nomogram (excluding the number of comorbidities). **(B)** The updated nomogram (including the number of comorbidities). The updated nomogram exhibits a larger gap between the density peaks of positive and negative events, indicating its superior ability to distinguish patients with and without poor HRQoL.
