# Supplementary figures and images for: Prediction of postoperative health-related quality of life among patients with metastatic spinal cord compression secondary to lung cancer
Source: Front Endocrinol (Lausanne). 2023 Sep 1;14:1206840. doi: 10.3389/fendo.2023.1206840 (PMC10502718; doi:10.3389/fendo.2023.1206840)

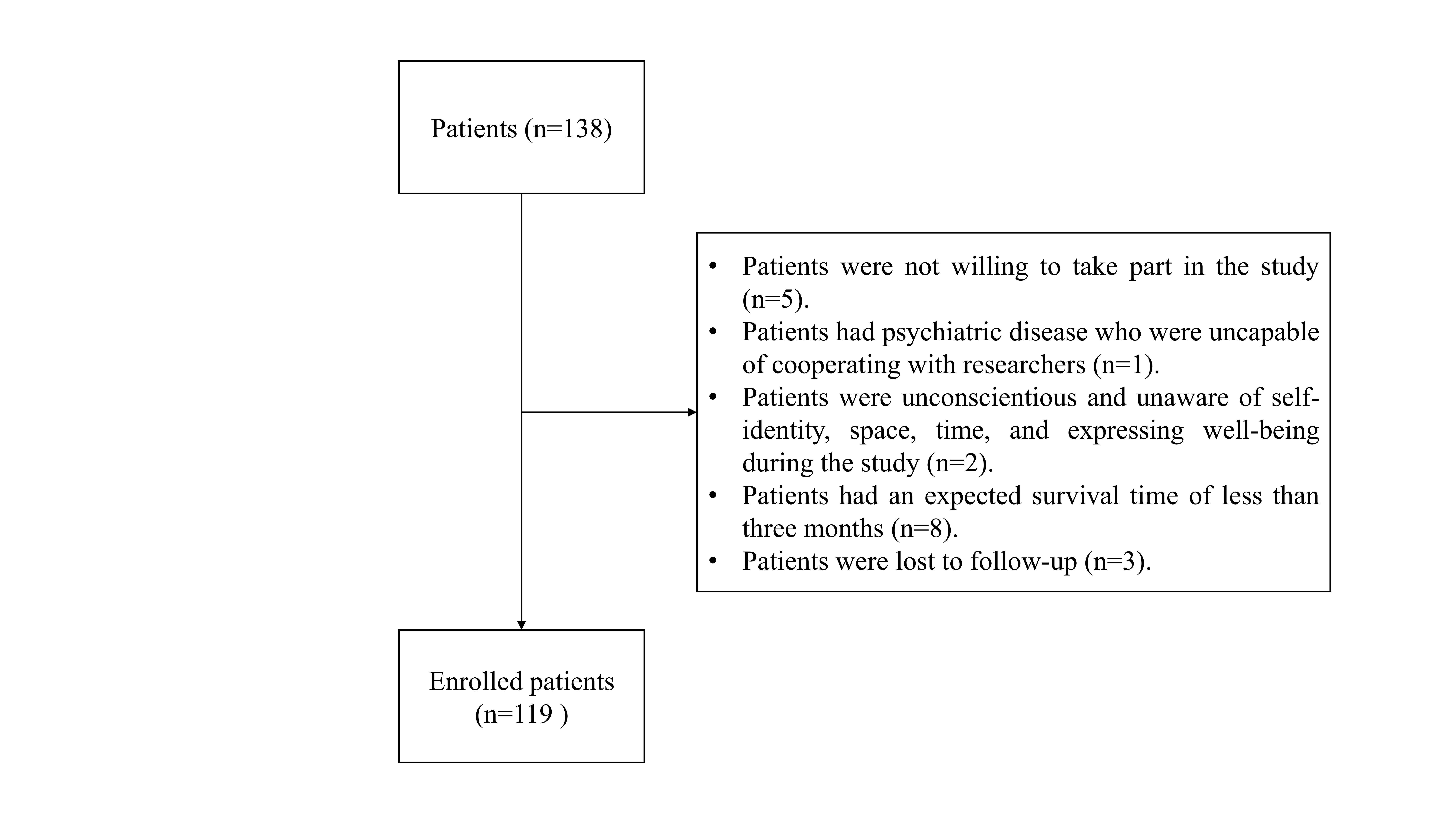

Supplement: Supplementary Figure 1 — Flowchart depicting the patient selection process. [file Image_1.tif]

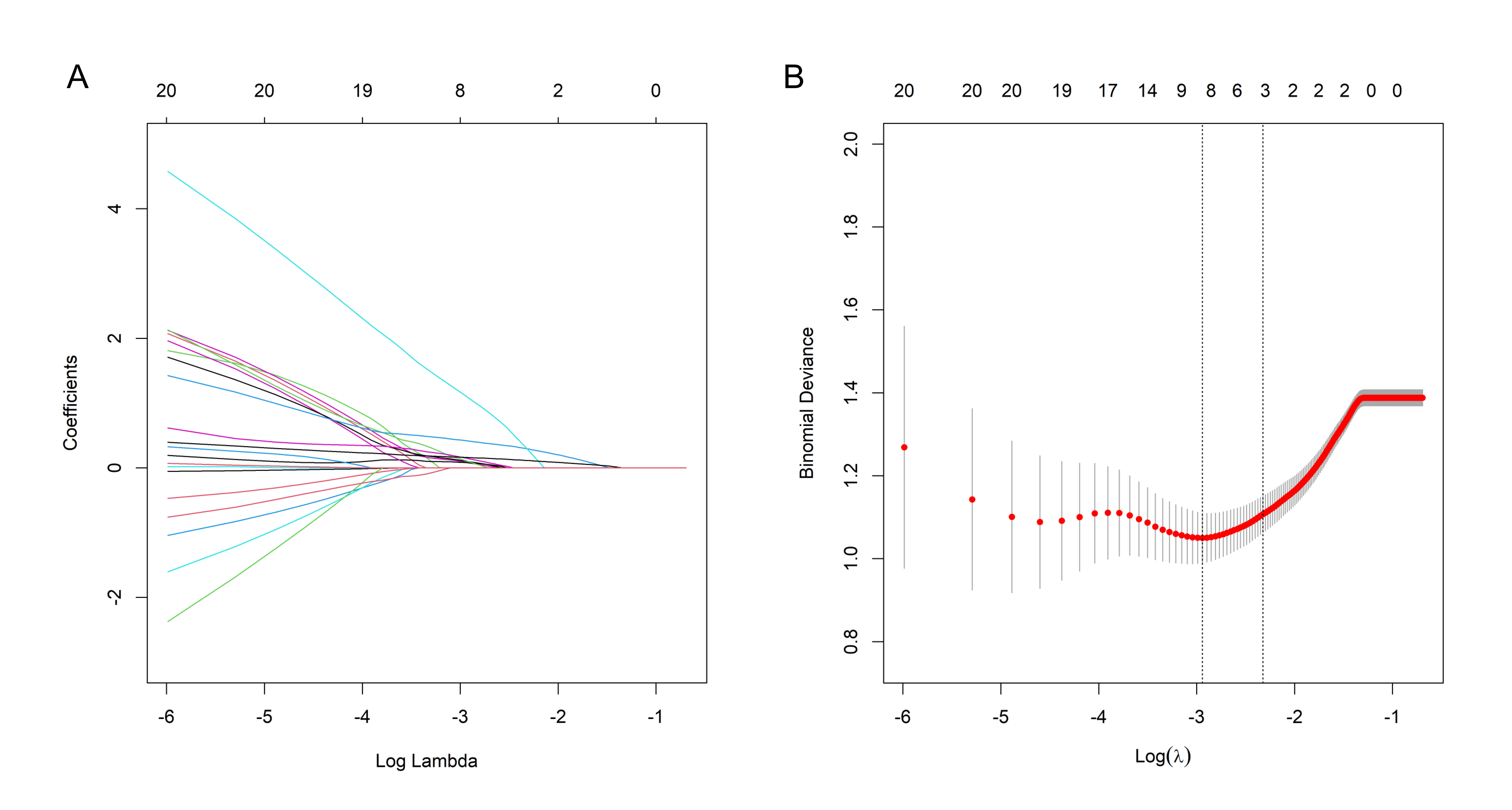

Supplement: Supplementary Figure 2 — Identification of variables using least absolute shrinkage and selection operator (LASSO) regression analysis combined with ten-fold cross-validation. (A) Coefficients of all variables based on the variable-screening method. (B) Selection of the appropriate log(λ) parameter based on the variable-screening method. The left dotted vertical line indicates the optimal value according to the minimum criteria, and the right dotted vertical line indicates one standard error of the minimum criteria. [file Image_2.tif]

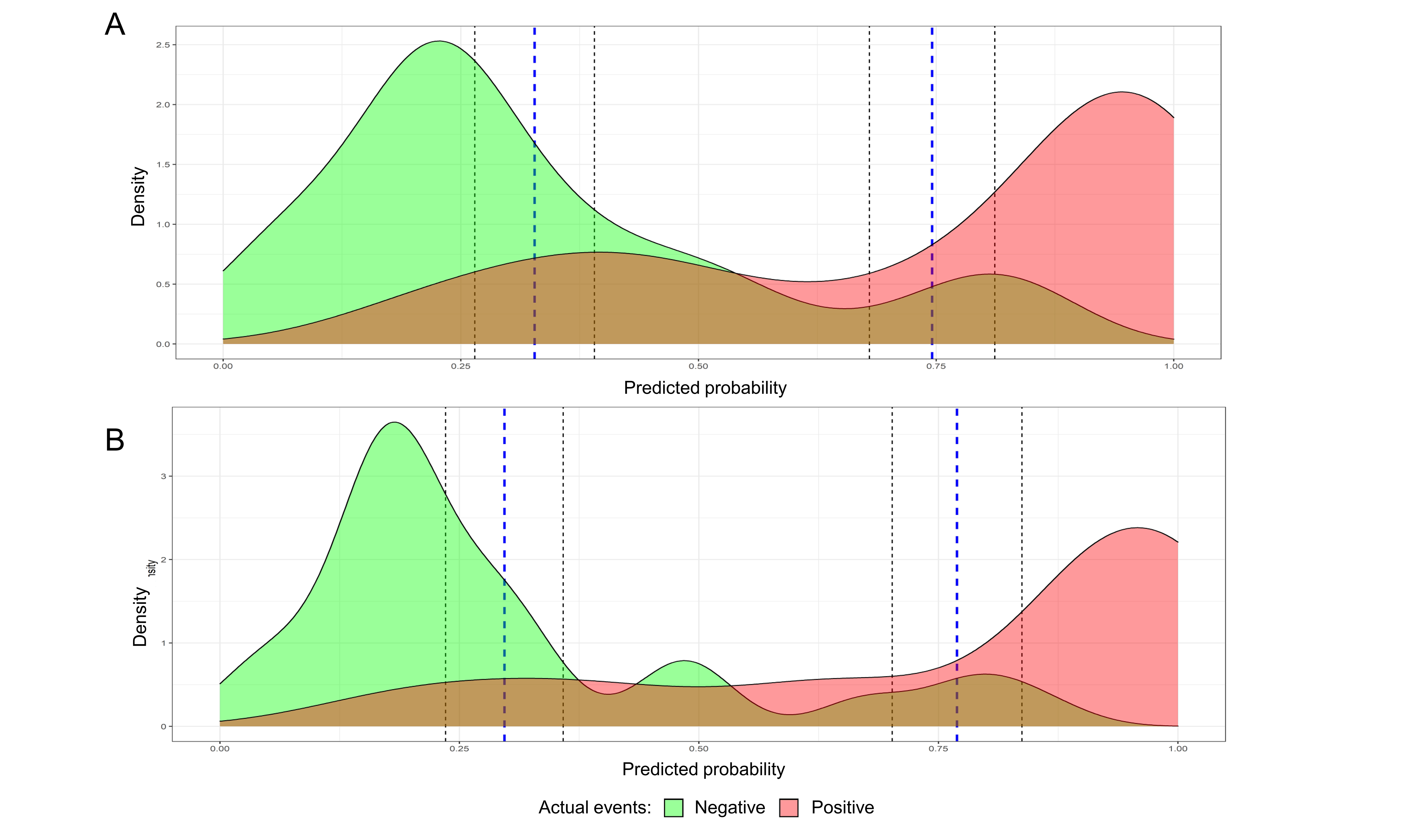

Supplement: Supplementary Figure 3 — Risk probability density curve for patients with and without poor HRQoL. (A) The previous nomogram (excluding the number of comorbidities). (B) The updated nomogram (including the number of comorbidities). The updated nomogram exhibits a larger gap between the density peaks of positive and negative events, indicating its superior ability to distinguish patients with and without poor HRQoL. [file Image_3.tif]
